# Occlusal Characteristics and Spacing in Primary Dentition: A Gender Comparative Cross-Sectional Study

**DOI:** 10.1155/2014/512680

**Published:** 2014-10-29

**Authors:** Madhuri Vegesna, R. Chandrasekhar, Vinay Chandrappa

**Affiliations:** Department of Pedodontics and Preventive Dentistry, Vishnu Dental College, Vishnupur, Bhimavaram, Andhra Pradesh 534202, India

## Abstract

*Context.* Occlusion in primary teeth varies among children of different populations and races. *Aim*. To assess and compare the occlusal characteristics and spacing in primary dentition among 3–6-year-old Dravidian children. *Materials and Methods*. The study included 2281 school going children. The primary molar relation, canine relation, overjet, and overbite were assessed using Foster and Hamilton criteria. Spacing conditions were registered according to Kisling and Krebs criteria. *Results.* The flush terminal plane molar relation (80.3%) was the most common primary molar relation. The distal step molar relation was more frequently found in female children (12.8%) than in males (8.6%). Class 1 canine relation was the most prevalent canine relation (81.3%) among males and females. Ideal overjet (84.3%) and overbite (72.7%) were observed among the majority of the children. Spaced type of arches occurred more frequently than closed arches in this sample. The incidence of primate spaces was more in males than in females. *Conclusion.* The study population has fewer deviations from normal occlusion which indicates decreased tendency for malocclusion in permanent dentition. However, further longitudinal studies are necessary to identify the potential limitations of a clinical approach relying on early orthodontic diagnosis and intervention.

## 1. Introduction

Childhood is the mirror in which the propensities of adulthood are reflected; similarly the type of occlusion in primary dentition predicts the occlusion of the permanent dentition [1]. The understanding of the anteroposterior changes that occur in the occlusion between the primary and permanent dentition is crucial for the clinicians involved in early orthodontic treatment [2]. Normal occlusion in primary teeth has the following characteristics: spacing between anterior teeth, primate spaces, low overjet and overbite, flush terminal plane molar relation, and ovoid arch form [3, 4]. The deviations in occlusion in primary dentition would be carried to succeeding permanent dentition and to a more pronounced degree [5].

Spacing is a common condition in the primary dentition and constitutes a very important feature of the dentition as it is an indicator of favorable development of permanent occlusion. Spacing often presents between all anterior primary teeth with the most marked spaces present being mesial to canines in the maxilla and distal to canines in the mandible. These are called primate spaces. The secondary or developmental spaces which are commonly found between the incisors are termed physiological spaces [6, 7]. The incidence of spacing in primary dentition varies from 42.9% to 98%. Perhaps lack of spacing suggests severe risk for crowding in the permanent dentition [6–8]. Spacing is more common in the maxilla than in the mandible and spaces are observed more among boys rather than girls [8].

Many observational studies relating to the spacing and occlusion of the primary dentition have confirmed that the occlusal characteristics vary among populations and ethnic groups. The present study documents the nature of occlusal relationships comprising molar relation, canine relation, overjet, overbite, and spacing of the primary dentition and also assesses gender variations among 3–6-year-old Dravidian children.

## 2. Materials and Methods

This cross-sectional study was conducted among a total of 2281 school children which included 1122 boys and 1159 girl aged 3–6 years with average age of 4.5 years. The study has been approved by the institutional ethical review board. Stratified cluster random sampling method was used to select children from the schools. The age of the child was obtained from school records. Children with complete set of primary dentition without any partially/completely erupted permanent teeth were included in the study, while those with extensive caries that affected the mesiodistal and occlusogingival dimension of teeth and infraocclusion and those with developmental anomalies were excluded from the study.

The children were examined in their respective schools by a single examiner under good day light. The primary molar relation, canine relation, overjet, and overbite were assessed using Foster and Hamilton criteria with the teeth in centric occlusion [9].

Terminal plane relationship of the second primary molars was evaluated and recorded as class 1: the distal surfaces of maxillary and mandibular primary second molars lie in the same vertical plan; class 2: the distal surface of the mandibular primary second molar is posterior to that of maxillary primary second molar; and class 3: the distal surface of the mandibular primary second molar is anterior to that of the maxillary primary second molar [9].

Primary canine relationship was evaluated and recorded as class 1: the tip of the maxillary primary canine tooth is in the same vertical plane as the distal surface of the mandibular primary canine; class 2: the tip of the maxillary primary canine tooth is mesial to the distal surface of the mandibular primary canine; class 3: the tip of maxillary primary canine is distal to the distal surface of the mandibular primary canine [9].

Overjet was measured as the greatest distance between the incisal edges of the maxillary and mandibular primary incisors in the occlusal plane using a millimeter gauge and recorded as ideal, if a positive overjet was less than or equal to 2 mm; increased, if it was greater than 2 mm; and reversed, if there was anterior cross-bite and edge-to-edge relationship was also assessed [9].

Vertical occlusion was graded according to the coverage of mandibular incisor by the most protruded fully erupted maxillary incisor and was recorded as ideal: if the lower primary incisal edges were contacting the palatal surfaces of the upper primary central incisors in centric occlusion, increased, if the mandibular incisors were touching the palate, open bite, when a gap existed between the incisal edges of incisors along the occlusal plane, and reduced, if the incisal tips of the lower primary incisors were not contacting the upper incisors or the palate in centric occlusion but with positive overbite [9].

Spacing conditions were registered between all teeth in the mandible and maxilla and graded according to Kisling and Krebs criteria: overlapping of teeth, contact, no contact, and space ≥2 mm. Dental floss was used to confirm the presence/absence of contacts, when doubtful [10].

*Statistics*. The obtained data was stored in excel sheet and analyzed using statistical software (SPSS version 16.0, Chicago). Chi-square test was used to compare the variables assessed within the population. For all tests a *P* value of ≤ 0.05 was set for statistical significance and a *P* value of ≤ 0.001 represented highly significant relation.

## 3. Results

Bilateral flush terminal plane molar relationship (80.3%) was the most prevalent molar relation while unilateral flush terminal plane with mesial step (2.3%) was the least common both in males and females. There was statistically significant difference (*P* ≤ 0.05) in molar relation among males and females (Table 1).

The most common type of canine relation was bilateral class 1 (81.3%) whereas the least frequent was unilateral class 1 with class 3 (2.7%); similar trend was observed in both genders (Table 2). Statistical significant difference was found among the sexes with respect to canine relation (*P* ≤ 0.05).

An ideal overjet was observed among 84.3% children followed by increased overjet (8.9%) and edge-to-edge bite (3.5%), while the least frequent type was reverse overjet (1.7%) (Table 3). The evaluation of overbite showed that 72.7% children had ideal overbite, 19.4% had increased bite while 1.5% had anterior open bite, and 1% had reduced bite (Table 4). Similar trend of prevalence was observed among both the sexes with respect to overjet and overbite. Statistical significant difference was found among the sexes with respect to overjet (*P* ≤ 0.05) while no significance was found with regard to overbite (*P* = 0.781).

The most frequent site of spacing (Table 5) in the maxillary arch coincided with the anthropoid space between the lateral incisor and canine (71.8%); however in mandibular arch it did not coincide with primate spaces; instead, spacing was found at two sites, that is, between canine and lateral incisor (31.1%) and lateral incisor and central incisor (31.0%). The spaces greater than or equal to 2 mm were found most commonly in relation to maxillary primate spaces (1.8%). The sites of contact of teeth were found most frequently between first and second primary molar in both maxilla (99.2%) and mandible (99.3%).

Statistical significant difference was found between the sexes (Table 6) with respect to spaces between first molar and canine (*P* ≤ 0.05), canine and lateral incisor (*P* ≤ 0.001), and central and lateral incisor (*P* ≤ 0.001).

## 4. Discussion

The occlusion of the primary dentition is completely established by the age of 3 years and lasts until about 6 years of age when the first permanent tooth begins to erupt [11]. Understanding the association between morphological aspects in the primary dentition and its transition to the permanent dentition provides the possibility of predicting the final permanent occlusion [12]. The anteroposterior relation of maxillary and mandibular permanent molars is an important criterion for recognition of malocclusions. This determines the necessity of interceptive orthodontics.

The flush terminal plane in the primary dentition is the most predominant primary molar relation followed by mesial and distal step [5, 6, 11, 13–18] and similar trend was noticed in our study. The flush terminal plane relation was frequently found in our population (80.3%); however distal step (10.7%) was more frequent than mesial step (3.6%). Yilmaz et al. in a cross-sectional study evaluated the occlusion in 205 3–6-year-old Turkish children and found that flush terminal plane was presented by 88.29% followed by distal step (7.31%) and mesial step (4.4%) [19]. Analogous observations were reported in Finnish and Iranian children [20, 21].

The flush terminal plane was found to be the most common molar relation and considered ideal for transition to class 1 in permanent dentition. However, mesial step was found to be the norm for completed primary dentition rather than flush terminal plane [22–24]. The magnitude of mesial step determines whether it would result in Angle's class I or class III molar relationship. Since flush terminal plane was the most common molar relationship in the present study we anticipate that the majority of the sample may have a favorable permanent molar relation.

The findings of the present study showed that 81.3% had class 1 canine relationship, 5.9% had class 2, and 5.8% had class 3. These findings were similar to the previous studies conducted in different populations [4, 11, 14, 16, 17, 19, 25]. Contradictory findings were reported in Finnish children among whom class II relation (52.4%) was the most prominent followed by class I (46.1%) and class III (1.5%) [20]. Canine and molar relationship together can be a diagnostic aid to predict changes in occlusal relationship. In clinical situations like flush terminal plane with class II canine relationship indicates a higher risk to develop a distal occlusion in permanent dentition. Therefore both the molar and canine relationships are taken into consideration to make a reliable prediction of the intermaxillary relationship in the permanent dentition [20].

The results of our investigation revealed ideal overjet in 84.3% children, increased overjet in 8.9%, edge-to-edge bite in 3.5%, and reversed overjet in 1.7% of the children. This finding is consistent with Nigerian children who demonstrated ideal overjet in 68.6%, increased overjet in 14.7%, edge-to-edge bite in 9.7%, and reverse bite in 7.0% [5]. Majority of Saudi and Chinese children had ideal overjet as seen in the present study [11, 26]. On the contrary, Foster and Hamilton found increased overjet in 72% of 2^1^/2-3-year-old children and Ravn [27] reported increased overjet in 27% of Copenhagen children [9].

An ideal overbite was found in 72.7% of children while 19.4% had increased bite, 1.5% had anterior open bite, and 1% had reduced bite. These findings were akin to those reported in Saudi Arabian children [11]. A study in Jordanian population demonstrated overbite in 44.3% children, 21.85% with reduced overbite, 5.7% with anterior open bite, and 28.2% with increased bite [7]. Anterior open bite was reported in 8% of African American children [23]. Majority of Chinese children had increased bite [26], whereas Belgian children exhibited open bite in 32% of the studied population [28]. Increased open bite could be attributed to oral habits such as dummy sucking and finger sucking [5]. Presently, no data indicates a definitive threshold value for overbite or overjet that could be applied in early diagnostics [20]. The higher prevalence of ideal overjet and overbite observed in our population may be conducive to achieve ideal anterior relation in permanent dentition.

Spaces in the primary teeth are described as physiological or developmental spaces. The spacing around the canines is termed as the simian gap [6], primate space [13], or anthropoid space [9], since they are prominent in dentitions of certain lower primates. Based on spacing between the teeth, Baume has classified the arrangement of primary dentition into two forms: open or type I and closed or type II [6].

Anterior spacing appears to be a common finding in our study population. Primate spaces were frequently found in the maxilla than mandible [3, 7, 29, 30] as observed in the present study. Male children demonstrated more frequency of primate spaces than females both in the maxilla and mandible. In contrast, primate spaces did not show significant difference between the genders in Tehran and Saudi children [21, 31]. Extreme spacing of ≥2 mm in our study was associated with anthropoid spaces in the maxilla (1.8%) akin to observations made by Otuyemi [5].

The incidence of crowding or overlapping of teeth in our sample was more prevalent in the mandible than in the maxilla. In mandible, crowding was predominant between the lateral incisor and canine (9.2%) and similar findings were reported in Nigerian children [5]. This study illustrated that crowding was more prevalent in females than in males in both maxilla and mandible. Majority of the study population demonstrated spaced arches which would lead to favorable permanent occlusion.

## 5. Conclusion

Occlusal characteristics vary among populations. To summarize, flush terminal plane, class I canine relation, ideal overjet, ideal overbite, and spaced arches prevailed among majority of the study population without any gender variations. These findings suggest favorable occlusal characteristics and spacing in primary dentition. However, future longitudinal studies are needed to observe whether the transition of these occlusal characteristics will lead to favorable occlusion in the permanent dentition.

## Figures and Tables

**Table 1:** Gender wise distribution of molar relation.

Type of molar relationship		Sex		Total
		Male	Female	
Flush terminal plane—bilateral	Count	916	916	1832
	% within SEX	**81.6%**	**79.0%**	**80.3%**
Distal step—bilateral	Count	97	148	245
	% within SEX	**8.6%**	**12.8%**	**10.7%**
Mesial step—bilateral	Count	43	39	82
	% within SEX	**3.8%**	**3.4%**	**3.6%**
Unilateral flush terminal plane with distal step	Count	37	30	67
	% within SEX	**3.3%**	**2.6%**	**2.9%**
Unilateral flush terminal plane with mesial step	Count	28	25	53
	% within SEX	**2.5%**	**2.2%**	**2.3%**
Unilateral mesial step with distal step	Count	0	0	0
	% within SEX	**0.0%**	**0.0%**	**0.0%**
Posterior cross-bite	Count	1	1	2
	% within SEX	**0.1%**	**0.1%**	**0.1%**

Total	Count	1122	1159	2281
	% within SEX	**100.0%**	**100.0%**	**100.0%**

*χ* ^2^ = 11.179, *P* = 0.025, significant.

**Table 2:** Gender wise distribution of canine relationship.

Type of canine relationship		Sex		Total
		Male	Female	
Class 1—bilateral	Count	918	936	1854
	% within SEX	**81.8%**	**80.8%**	**81.3%**
Class 2—bilateral	Count	78	56	134
	% within SEX	**7.0%**	**4.8%**	**5.9%**
Class 3—bilateral	Count	50	82	132
	% within SEX	**4.5%**	**7.1%**	**5.8%**
Unilateral class 1 with class 2	Count	50	46	96
	% within SEX	**4.5%**	**4.0%**	**4.2%**
Unilateral class 1 with class 3	Count	24	38	62
	% within SEX	**2.1%**	**3.3%**	**2.7%**
Unilateral class 2 with class 3	Count	1	0	1
	% within SEX	**0.1%**	**0.0%**	**0.0%**
Posterior cross-bite	Count	1	1	2
	% within SEX	**0.1%**	**0.1%**	**0.1%**

Total	Count	1122	1159	2281
	% within SEX	100.0%	100.0%	100.0%

*χ* ^2^ = 15.301, *P* = 0.009, significant.

**Table 3:** Distribution of overjet variations.

Overjet		Sex		Total
		Male	Female	
Ideal	Count	949	975	1924
	% within SEX	**84.6%**	**84.1%**	**84.3%**
Increased	Count	111	91	202
	% within SEX	**9.9%**	**7.9%**	**8.9%**
Edge-to-edge	Count	33	47	80
	% within SEX	**2.9%**	**4.1%**	**3.5%**
Reversed	Count	11	27	38
	% within SEX	**1.0%**	**2.3%**	**1.7%**
Others	Count	18	19	37
	% within SEX	1.6%	1.6%	1.6%

Total	Count	1122	1159	2281
	% within SEX	100.0%	100.0%	100.0%

*χ* ^2^ = 10.986, *P* = 0.027, significant.

**Table 4:** Distribution of overbite variations.

Overbite		Sex		Total
		Male	Female	
Ideal	Count	826	832	1658
	% within SEX	**73.6%**	**71.8%**	**72.7%**
Increased	Count	225	217	442
	% within SEX	**20.1%**	**18.7%**	**19.4%**
Anterior open bite	Count	16	18	34
	% within SEX	**1.4%**	**1.6%**	**1.5%**
Reduced	Count	9	13	22
	% within SEX	**0.8%**	**1.1%**	**1.0%**
Others	Count	46	79	125
	% within SEX	**4.1%**	**6.8%**	**5.5%**

Total	Count	1122	1159	2281
	% within SEX	100.0%	100.0%	100.0%

*χ* ^2^ = 1.756, *P* = 0.781, not significant.

**Table 5:** Arch wise prevalence of spacing.

Site		Normal contacts	No contacts	Overlapping	Spacing ≥ 2 mm	*χ* ^2^	Significance *P* value
Second molar—first molar	Maxilla	4531	30	0	1	1.158	0.763 Not significant
		99.2%	0.7%	0%	0.1%		
	Mandible	4534	27	0	1		
		99.3%	0.6%	0%	0.1%		

First molar—canine	Maxilla	3938	624	0	0	198.963	0.001 Highly significant
		86.3%	13.7%	0%	0%		
	Mandible	3406	1152	2	2		
		74.6%	25.2%	0.1%	0.1%		

Canine—lateral incisor	Maxilla	1270	3190	16	86	1668.084	0.001 Highly significant
		27.8%	70.0%	0.4%	1.8%		
	Mandible	2736	1396	420	10		
		59.7%	30.9%	9.2%	0.2%		

Lateral incisor—central incisor	Maxilla	2853	1649	52	8	99.306	0.001 Highly significant
		62.5%	36.2%	1.1%	0.2%		
	Mandible	2959	1413	187	3		
		64.8%	30.9%	4.1%	0.1%		

Between central incisors	Maxilla	1779	485	8	9	124.711	0.001 Highly significant
		38.9%	10.7%	0.2%	0.2%		
	Mandible	1519	660	97	5		
		33.2%	14.5%	2.2%	0.1%		

**Table 6:** Arch wise and gender wise prevalence of spacing.

Site		Normal contacts		No contacts		Overlapping		Spacing > 2 mm		Significance [among males and females] *P* value
		Male	Female	Male	Female	Male	Female	Male	Female	
Second molar—first molar	Maxilla	2228	2304	16	14	0	0	0	0	*χ* ^2^ = 1.270 *P* = 0.530 Not significant
		99.1	99.4	0.9	0.6	0	0	0	0	
	Mandible	2234	2300	10	17	0	1	0	0	
		99.4	99.1	0.6	0.8	0	0.1	0	0	

First Molar—canine	Maxilla	1926	2012	318	306	0	0	0	0	*χ* ^2^ = 9.077 *P* = 0.028 Significant
		86.1	86.3	13.9	13.7	0	0	0	0	
	Mandible	1625	1781	317	536	1	1	1	0	
		72.4	76.8	27.4	23.1	0.1	0.1	0.1	0	

Canine—lateral incisor	Maxilla	568	701	1630	1561	6	10	40	46	*χ* ^2^ = 38.799 *P* = 0.001 Highly significant
		25.4	30.2	72.5	67.4	0.2	0.5	1.9	1.9	
	Mandible	1286	1450	770	626	184	236	4	6	
		57.0	62.4	34.6	27.1	8.2	10.2	0.2	0.3	

Lateral incisor—central incisor	Maxilla	1329	1424	880	769	34	17	1	8	*χ* ^2^ = 16.304 *P* = 0.001 Highly significant
		59.6	65.4	38.8	33.5	1.5	0.8	0.1	0.3	
	Mandible	1438	1421	714	699	90	97	2	1	
		64.0	65.6	31.8	30.2	4.1	4.1	0.1	0.1	

Between central incisors	Maxilla	883	896	232	253	6	2	1	8	*χ* ^2^ = 5.480 *P* = 0.140 Not significant
		78.7	77.3	20.7	21.8	0.5	0.2	0.1	0.7	
	Mandible	734	785	348	312	38	59	2	3	
		65.4	67.7	31.0	26.9	3.4	5.1	0.2	0.3	
